# Experimental Analysis of NiTi Alloy during Strain-Controlled Low-Cycle Fatigue

**DOI:** 10.3390/ma14164455

**Published:** 2021-08-09

**Authors:** Pedro Cunha Lima, Patrícia Freitas Rodrigues, Ana Sofia Ramos, José D. M. da Costa, Francisco Manuel Braz Fernandes, Maria Teresa Vieira

**Affiliations:** 1 University of Coimbra, CEMMPRE, Department of Mechanical Engineering, 3030-790 Coimbra, Portugal; pedrolima@ifba.edu.br (P.C.L.); sofia.ramos@dem.uc.pt (A.S.R.); jose.domingos@dem.uc.pt (J.D.M.d.C.); teresa.vieira@dem.uc.pt (M.T.V.); 2 Instituto Federal de Educação, Ciência e Tecnologia da Bahia—IFBA, Salvador 40301-015, Brazil; 3 CENIMAT/I3N, Materials Science Department, NOVA School of Science and Technology, Universidade NOVA de Lisboa, 2829-516 Caparica, Portugal; fbf@fct.unl.pt

**Keywords:** NiTi, shape memory alloy, stress-induced martensite, resistivity

## Abstract

The interaction between the stress-induced martensitic transformation and resistivity behavior of superelastic NiTi shape memory alloy (SMA) was studied. Strain-controlled low-cycle fatigue up to 6% was monitored by in situ electrical resistivity measurements. The experimental results show that a great motion of martensite fronts results in a significant accumulation of defects, as evidenced by transmission electron microscopy (TEM), before and after the tensile cycles. This gives rise to an overall increase of the resistivity values up to the maximum deformation. Therefore, the research suggests that shape memory alloy wire has great potential as a stress sensor inside bulk materials.

## 1. Introduction

Shape memory alloy (SMA) phase transformations can be used to identify environmental changes around them and react immediately to external stimuli. SMAs have interesting characteristics such as large deformation ability, as well as actuating function, in particular, where SMA structures are subjected to cyclic loading [1,2]. Among the SMAs, the most investigated one is the NiTi alloy due to its peculiar functional properties—superelasticity and shape memory effect. The superelasticity is observed as a result of the stress-induced martensitic transformation (SIM) between austenite and oriented martensite [3,4].

When loaded under isothermal conditions, SMAs first show the elastic deformation in the austenite phase, followed by a maximum plateau associated with the stress-induced transformation from austenite (A) to martensite (M). Upon unloading, the material undergoes the reverse phase transformation from martensite to austenite, which occurs at lower values of stress plateau than those observed in forward transformation. The plastic deformation affects the SMAs superelastic cyclic behavior due to the local stress field created at the phase transformation fronts, promoting dislocation slips, which corresponds to irreversible deformations in A-M interfaces. The internal stress field supports the nucleation of martensite variants. Therefore, the residual martensite (associated with the accumulated residual strain during cycling) is responsible for a reduction of the critical stress to promote the phase transformation and decreases the hysteresis loop area during subsequent cycles [3]. For the strain-controlled loading, the degradation mechanism occurs due to the accumulation of the residual strain that gives rise to a reduction of the recoverable strain [4,5].

The electrical resistivity of SMA wires may be evaluated during mechanical or even thermomechanical tests to detect the onset and end of the stress-induced martensitic transformation and/or reorientation processes. According to the state of the art, the mechanical tests performed (isothermic) suggest that the stress–strain behavior can be related to the mechanisms of the variant reorientation induced by the stress state. In addition, during the thermoelastic martensitic transformation, there is an excellent linear relationship between the electrical resistivity and the strain [6,7,8,9]. The relative change of electrical resistivity versus strain reflects the structural evolution of the NiTi during tensile tests such as defect density upon tensile loading, e.g., the occurrence of austenite twins in the microstructure of deformed NiTi during the tensile test. Furthermore, the correlation between the electrical resistivity and applied strain suggests the potential of NiTi alloys as sensors inserted in parts subjected to cyclic loadings. The objective could be to detect cracks and contribute to the self-healing of metallic materials [10].

However, many aspects need a deeper analysis, in particular why the hysteretic stress-induced phase transformations significantly affect the damage mechanisms occurring under cyclic tensile loadings. Moreover, it should also be investigated how these transformations can be reflected in the resistivity behavior during the isothermal cyclic tensile load/unload tests [3,11,12,13,14,15,16].

The aim of the present work was to explore the damage mechanisms by studying the structural and functional characteristics of NiTi wires and their influence on electrical resistance. Thus, 300 cyclic tensile loading tests (low-cycle fatigue) were performed on a superelastic NiTi SMA wire, while measuring the electrical resistance. In the available literature, electrical resistivity measurements at constant temperature (room temperature) up to a similar number of cycles in NiTi alloys have not been reported. Ex situ TEM was carried out to analyze the deformation/transformation processes at controlled deformation.

## 2. Materials and Methods

### 2.1. Material

**Figure 1:**
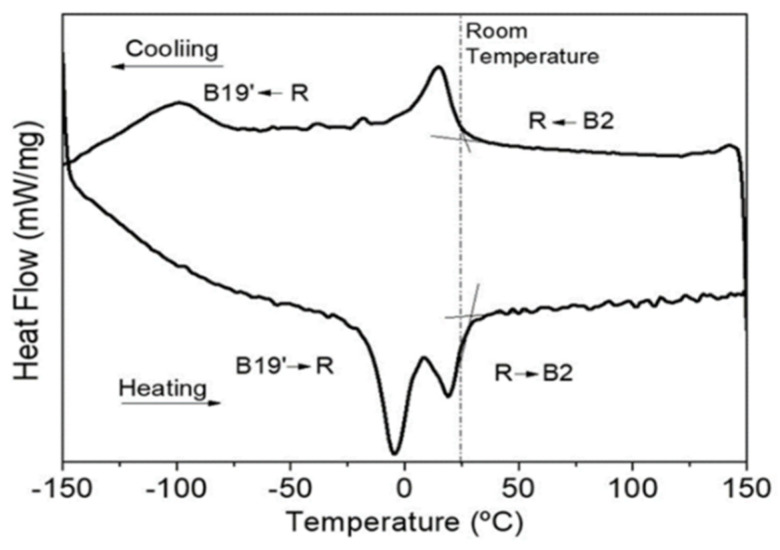
DSC results for NiTi wire as-received condition.

A superelastic NiTi wire (Fort Wayne Metals-FWM #4-50.7 at % Ni) with a diameter of 0.5 mm was selected for this investigation. The wire is a mixed structure of austenite and R phase at room temperature (25 °C), as shown by DSC (differential scanning calorimetry). Measurements were carried out at temperatures ranging from −150 °C to 150 °C under a controlled heating/cooling rate of 10 °C/min. Before examination by DSC, the samples (~15 mg) were cut and then chemically etched (10 vol% HF + 45 vol% HNO_3_ + 45 vol% H_2_O) in order to remove the oxide, as well as the layer deformed by the cutting operation (final mass: 17 mg). The results are shown in Figure 1. The phase transformation temperatures were determined: R_s_ = 25.9 °C; R_f_ = −10.0 °C; M_s_ = −76 °C; M_f_ = −110 °C; A_s_ = −16.2 °C and A_f_ = 27.9 °C. The as-received wire condition also was verified by the electrical resistivity test, which confirmed a mixture between austenite and R phase values at 8.36 × 10^−7^ Ω·m, without applied stress [2,17].

### 2.2. Experimental Conditions

The NiTi wire functional characteristics were studied in 300 complete tensile cycles at room temperature, during which the electrical resistivity was measured; particularly, the onset of the stress-induced martensitic transformation (σ_s_^AM^) was evaluated. The evolution of global stress–strain, as well as stress, strain and electrical resistivity as a function of time, was measured during the 300 cycles at room temperature. The tensile loading/unloading tests (Instron Eletroplus 10000E testing machine) were performed at a deformation rate of 1 mm/min, up to 6% strain using a gauge length of 44 mm. In order to avoid the introduction of another parameter, such as the temperature, a strain-controlled mode at a frequency of ~0.003 Hz was adopted.

The in situ electrical resistivity was performed along the 300 cycles. The four-probe method is one of the most used methods for the accurate measurement of resistivity [18]. The configuration applied was carefully projected to remove the influence of contact resistance on NiTi and electrical probes. The precision of the electrical resistivity measurement was ±1 × 10^−9^ Ω·m.

The phases and the microstructural defects created by tensile deformation were identified by transmission electron microscopy (TEM, FEI, Hillsboro, OR, USA) of samples of the wire cut from both commercial condition (unloaded) and after 300 superelastic cycles. TEM tests were performed using a FEI Tecnai G2 SuperTWIN FEG microscope operated at 200 kV. Selected area electron diffraction (SAED) patterns were acquired with a camera length of 860 mm and indexed with the lattice constant of the B2 austenite (a = 0.3015 nm) and the lattice constant of the B19′ martensite (a = 0.2889 nm, b = 0.4120, c = 0.4622 e β = 96.8°) [19]. The images and the SAED patterns were recorded with the use of the SIS MegaView camera. Process Diffraction^®^ software was used for the indexation of the SAED patterns. The samples for TEM investigations were cut using a FEI Quanta 3D 200 focused ion beam (FIB) equipment.

## 3. Results

### 3.1. Functional Behavior

**Figure 2:**
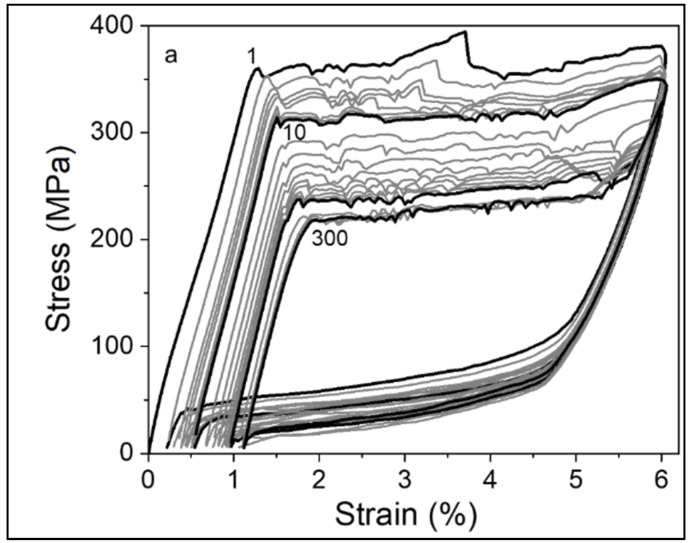
Stress-strain superelastic behavior up to 300th loading/unloading cycle of the NiTi (SMA wire) at room temperature.

Since the cyclic deformation of the NiTi wire promotes the accumulation of residual deformation during the first mechanical cycles, the analysis of low-cycle fatigue behavior was performed up to the 300th cycle. The main objective is to analyze the loss of functional properties of the wire (Figure 2) and to correlate it to the electrical resistivity variation. Some parameters vary along the tensile cycles, such as maximum stress plateau (direct transformation), minimum stress plateau (reverse transformation), and non-recoverable transformation (Figure 2) [15,17,20].

**Figure 3:**
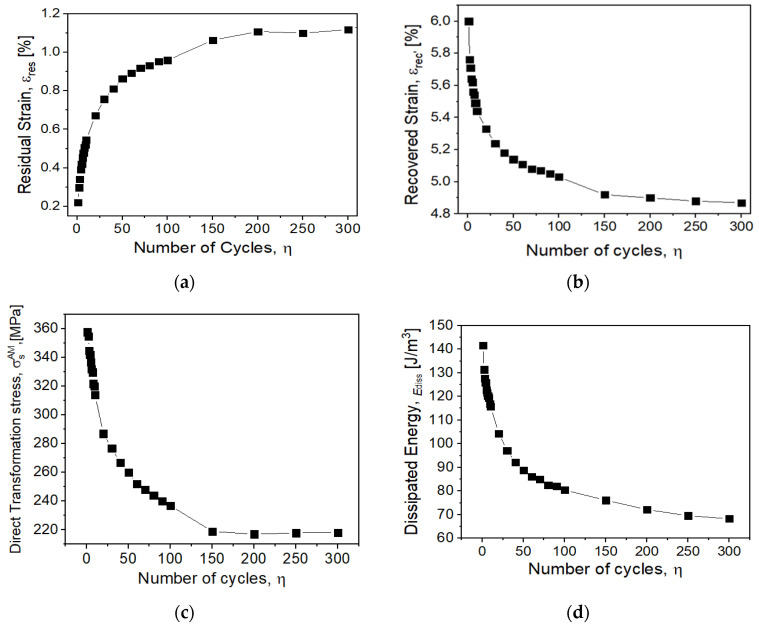
Mechanical behavior of NiTi wire. (**a**) Residual strain; (**b**) recovered strain; (**c**) direct transformation stress and (**d**) dissipated energy versus function of the number of cycles.

The difference in stress–strain hysteresis behavior, which involves stress-induced transformations, can be attributed to several mechanisms, as follows: (i) formation of stabilized martensite [5], (ii) detwinning [21], (iii) grain reorientation [3], (iv) shear deformation [22] and (v) formation of lattice defects [5]. Upon repeated cycle loadings, several preferentially oriented martensite variants achieve conditions that inhibit the possibility to recover the pristine condition. This results from increasing dislocation density due to shear deformation at B2–B19′ interfaces [23]. This condition is shown through the initial loop deformation value (ε_i_) at the end of the first cycle ε_i_ = 0.2% and for the 300th cycle ε_i_ = 1.2%. Figure 3 displays the correlation between transformation reversibility degradation and corresponding loss of functional properties. Figure 3a shows the gradual degradation of functional properties along the 300 cycles, by the trend of the residual strain. Figure 3b emphasizes the trend of the recovered strain. When the number of cycles increases, the influence on the transformation is more apparent; the content of austenite to be transformed is reduced due to the residual martensite present. After SIM, the residual martensite is not involved in the phase transformation, which can cause a reverse phase transformation degradation. As the number of cycles increases, the residual strain increases, while decreasing the recoverable strain and direct transformation stress (Figure 3a–c). The internal stress may explain this behavior due to the presence of dislocations and corresponding strain induced in neighboring grains; therefore, a part of the material remains in martensitic condition after unloading. These microstructural evolutions promote a gradual loss of functional properties [20]. Functional degradation of the material can be observed, but still keeping superelastic characteristics. However, an amount of non-recoverable transformation was already confirmed by recovered strain evolution (Figure 3b). Figure 3c shows the evolution of the direct transformation stress, σ_s_^AM^. This property exhibits a sharp decrease in the initial cycles (up to the 20th cycle), then it continues to decrease at a slower rate and stabilizes after 150 cycles. This behavior may be attributed to multiple microstructural mechanisms occurring during martensite phase formatio: (i) the accumulated damage stabilization along with the deformation cycles, (ii) the grain reorientation and (iii) the shear deformation [8]. The heterogeneous structure can promote a decrease in the critical stress to transform the austenite into martensite, and dislocation density can affect the internal stresses, which can assist the SIM transformation [5,13,24]. The dissipated energy shown in Figure 3d is related to the hysteresis in the mechanism of the SIM transformation, which decreases and converges to a stable value with increasing number of cycles. The change in the superelastic loop is noticeable for cycle 150, where it is possible to identify the $E_{diss}$ stabilization [2,25]. The highest reduction rate of the dissipated energy may be attributed to the plastic deformation and the increase of the dislocation density. The dislocations might lead to the formation of stress fields and, subsequently, to the development of stabilized stress-induced martensite. This behavior shows the influence of the B19′ deformation twinning in functional low-cycle fatigue of NiTi wire [5]. The stabilized values of $E_{diss}$ are about 25% of the first cycle value. All the characteristics (Figure 3) show noticeable changes up to the 150th cycle; this behavior suggests the possibility of fatigue crack initiation [21].

### 3.2. In Situ Electrical Resistivity versus Low-Cycle Fatigue

**Figure 4:**
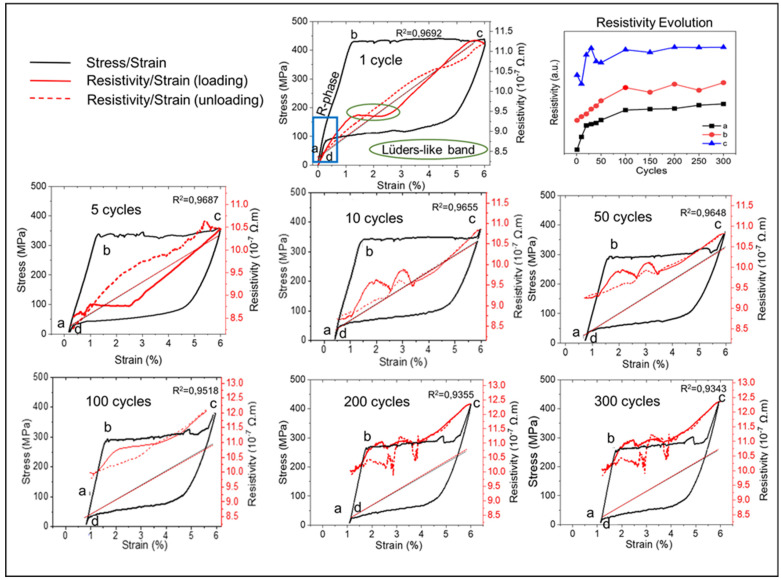
Evolution of stress-deformation (black curves) and resistivity-deformation (red curves) of the NiTi wire in the superelastic low-cycle fatigue test: 1st, 5th, 10th, 50th, 100th, 200th and 300th cycles (a-beginning of each load/unload cycle; b-beginning of the phase transformation plateau; c-end of the phase transformation plateau; d-end of each cycle).

The values and the behavior of the electrical resistivity measurements in uniaxial deformation of 1st, 5th, 10th, 50th, 100th, 200th and 300th cycles among 300 cycles are summarized in Table 1 and in Figure 4, respectively. There is a slight decrease in the maximum stress plateau value during the tensile cycles where SIM transformation occurs. This behavior is also observed in the electrical resistivity values at 6% of deformation, where an increase of about 9% is observed between the first cycle (11.21 *×* 10^−7^ Ω·m) and the 300th cycle (12.35 *×* 10^−7^ Ω·m).

Figure 4 shows that the resistivity has a linear behavior in the deformation direction. The applied strain increases the resistivity values; the curve of resistivity–deformation is linear with stress–deformation curves (Figure 4). This behavior is related to the mechanism observed during the low-cycle fatigue test performed. During SIM, the electrical resistivity increases with strain as a consequence of the lattice defects involved. The amount of B19′ grows linearly with deformation and both phases (B2 and B19′) must be considered, then the resistivity must be the linear combination of the contribution of each phase.

The resistivity changes with the applied strain during the stress-induced transformation plateau, while small changes are observed during elastic deformation. This behavior confirms that the resistivity of the shape memory alloy (NiTi) is a function of the volume fraction of each phase [26]. Figure 4 shows that the stress applied is high enough to induce the B2–B19′ transformation, which occurs with an observation of the R phase presence due to a change in the resistivity values measured (blue square in first cycle) [27].

The beginning (close to 1%, marked by b) and the end part (at 6%, marked by c) of the SIM transformation plateau, associated with the transformation B2 → B19′, is as evidenced by the resistivity change (Figure 4). Taking into account that there is austenite to transform and the reorientation of the martensite associated with the Lüders-like deformations, this change is probably related to the coalescence of one or more bands of Lüders-like transformation [28,29]. The resistivity plateau at the first cycle (green circle) between 1.25% and 2.5% strain can be associated with the Lüders-like band stabilization during SIM transformation. Similar behavior was not observed in the 300th cycle. The residual martensite formed along the low-cyle fatigue test was observed and confirmed by resistivity values.

Although the resistivity behavior is predictable, as desired, it presents changes related to the transformation. The resistivity at the beginning of each new cycle (point a in Figure 4) is continuously increasing with increasing number of cycles (Table 1, Figure 4). The values ranging from 8.2 × 10^−7^ Ω·m to 10.0 × 10^−7^ may indicate the presence of mixed-phase B2 and B19′, but the superelastic behavior of the material is still preserved.

Since the resistivity depends on the volume fractions of the martensitic and austenitic phases (Figure 4 (points a, b, c and d) and Table 1), a linear relationship is observed between the resistivity and strain. Usually, the resistivity–strain curve does not show a significant hysteresis [26]. This behavior allows the use of the resistivity values as a parameter of strain variation.

### 3.3. TEM Analysis

**Figure 5:**
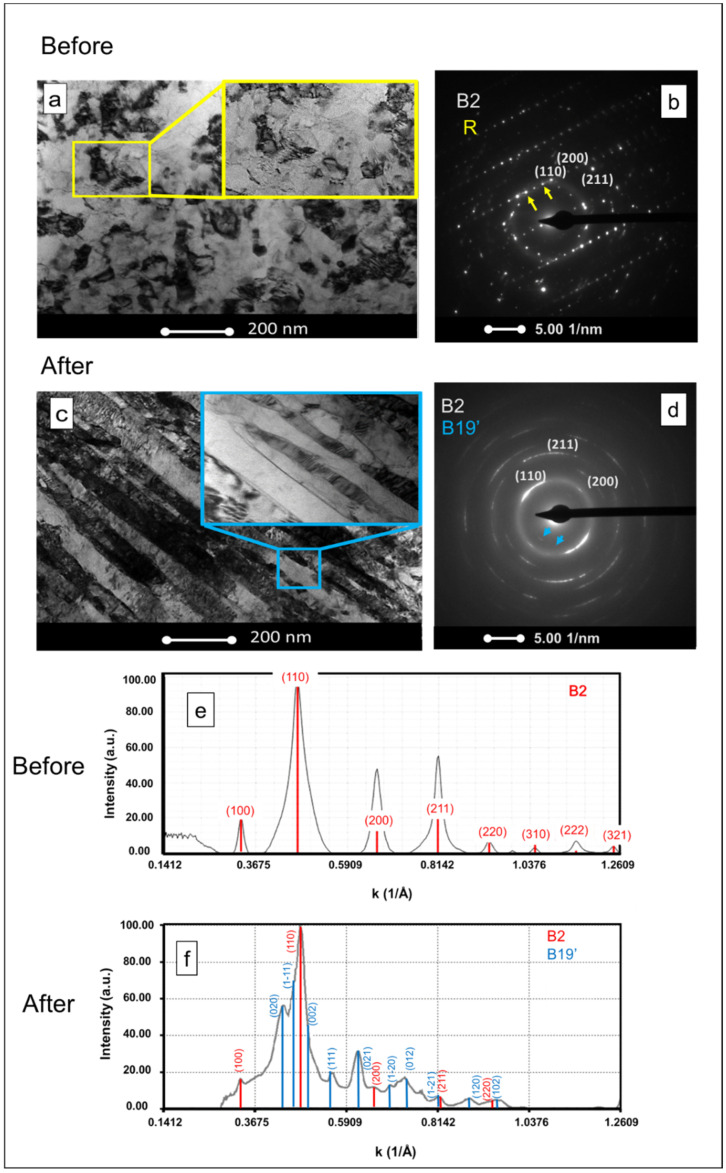
Microstructures of NiTi after and before low-cycle fatigue tests (TEM); (**a**,**c**) BF, (**b**,**d**) SAED patterns and (**e**,**f**) respective indexation.

Microstructures before and after the low-cycle fatigue test were analyzed by TEM. The bright-field (BF) image before deformation (Figure 5a) consists of homogeneously distributed equiaxed grains of different sizes. It can be observed that all grains have dimensions in the range of a few hundred nanometers. In the as-received wire, it is possible to observe (110), (200) and (211) reflections corresponding to the B2 NiTi phase (Figure 5b), as well as the superlattice appearance of reflections in the 1/3 position along the direction <110>, indicated by the yellow arrows. These reflections are characteristic of the R phase [30]. Figure 5c shows the microstructure after the cyclic tensile test. The BF image reveals a lamellar microstructure with band widths ranging between 50 and 80 nm, which confirms that plastic deformation occurred. Inside the bands, there were even smaller twin lamellae, suggesting the presence of stress-induced B19′ (blue square). SAED pattern (Figure 5d) shows the presence of the B19′ phase. This phase is represented by the weak diffraction points highlighted by the blue arrows. In addition, it is possible to observe incomplete rings corresponding to (110), (200) and (211) reflections of the B2 phase. These results are corroborated by the recovered strain values (Figure 3b) and the resistivity behavior (Table 1), which indicate a mixed-phase structure after the low-cycle fatigue tests. The indexation of the diffraction patterns indicates the presence of B2 before deformation (Figure 5e), and a mixed-phase structure of B2 and B19′ after deformation (Figure 5f). The TEM analysis also confirms the presence of dislocations as a result of the deformation process after tensile testing [31].

## 4. Conclusions

The present study contributes to establish the low-cycle fatigue behavior at room temperature of NiTi wires, as summarized below:

- The functional degradation as evidenced by the residual strain evolution is the result of untransformed austenite and remaining martensite formed during cycling; more B2–B19′ interfaces and a higher dislocation density are present in the material as the number of cycles progresses;
- The electrical resistivity increases linearly with strain; the electrical resistivity at the beginning of each cycle increases with the number of cycles, revealing the presence of a mixture of phases (B2 and B19′);
- During cycling, the resistivity at the beginning of each new loading cycle, as well as the resistivity at the beginning of each upper SIM plateau, gradually change; this shows that NiTi may be effectively used as a crack propagation sensor when inserted inside a component subjected to low-cycle fatigue.

## Figures and Tables

**Table 1:** Evolution of the stress, strain and resistivity values of the NiTi wire corresponding to points a, b, c and d.

	Stress (MPa)				Strain (%)				Resistivity (10^−7^ Ω·m)			
Cycle/Points	a	b	c	d	a	b	c	d	a	b	c	d
1	0.00	362.00	386.00	0.00	0.00	1.25	6.00	0.20	8.16	9.36	11.21	8.29
5	0.00	328.10	349.80	0.00	0.34	1.37	6.00	0.38	8.28	8.77	10.42	8.29
10	0.00	314.00	349.01	0.00	0.50	1.68	6.00	0.50	8.68	9.51	10.85	9.05
50	0.00	310.80	406.92	0.00	0.80	1.72	6.00	0.90	9.37	10.16	11.72	9.37
100	0.00	237.00	321.00	0.00	0.97	1.96	6.00	1.00	9.78	10.70	12.25	9.83
200	0.00	217.00	319.00	0.00	1.14	2.00	6.00	1.12	9.84	10.84	12.35	9.85
300	0.00	216.00	327.00	0.00	1.20	2.15	6.00	1.25	10.03	10.90	12.35	10.04
